# ggEDA: Visualisations for exploratory data analysis using tiled one-dimensional graphics and parallel coordinate plots

**DOI:** 10.12688/f1000research.168305.1

**Published:** 2025-11-13

**Authors:** Sam El-Kamand, Julian M.W. Quinn, Mark J. Cowley

**Affiliations:** 1 Computational Biology, Children's Cancer Institute Australia, Sydney, New South Wales, 2052, Australia; 2 School of Clinical Medicine, University of New South Wales, Sydney, New South Wales, 2052, Australia

**Keywords:** R, visualisation, exploratory data analysis, multidimensional, parallel coordinate plots

## Abstract

Exploratory data analysis (EDA) involves summarising trends within a dataset to help uncover data quality issues and generate hypotheses. However, identifying relationships between multiple features often requires extensive coding, manual inspection and statistical modelling. Here, we introduce the ggEDA R package, which streamlines multidimensional data exploration by providing two turnkey and complementary visualisation strategies. ggEDA generates interactive parallel coordinate plots (PCPs) well suited for examining large datasets with mostly quantitative features, and introduces tiled one-dimensional plots that more effectively show missingness and reveal categorical relationships in smaller datasets. ggEDA reduces the amount of code and time required to detect multi-feature relationships that may otherwise require statistical modelling or thorough manual review to identify. To make ggEDA visualisations accessible to a wider audience we also developed interactiveEDA, a web app that enables non-programmers to explore and interpret data patterns interactively. ggEDA and interactiveEDA are available at https://github.com/CCICB/ggEDA and https://github.com/CCICB/interactiveEDA respectively.

## Introduction

**Figure 1.**
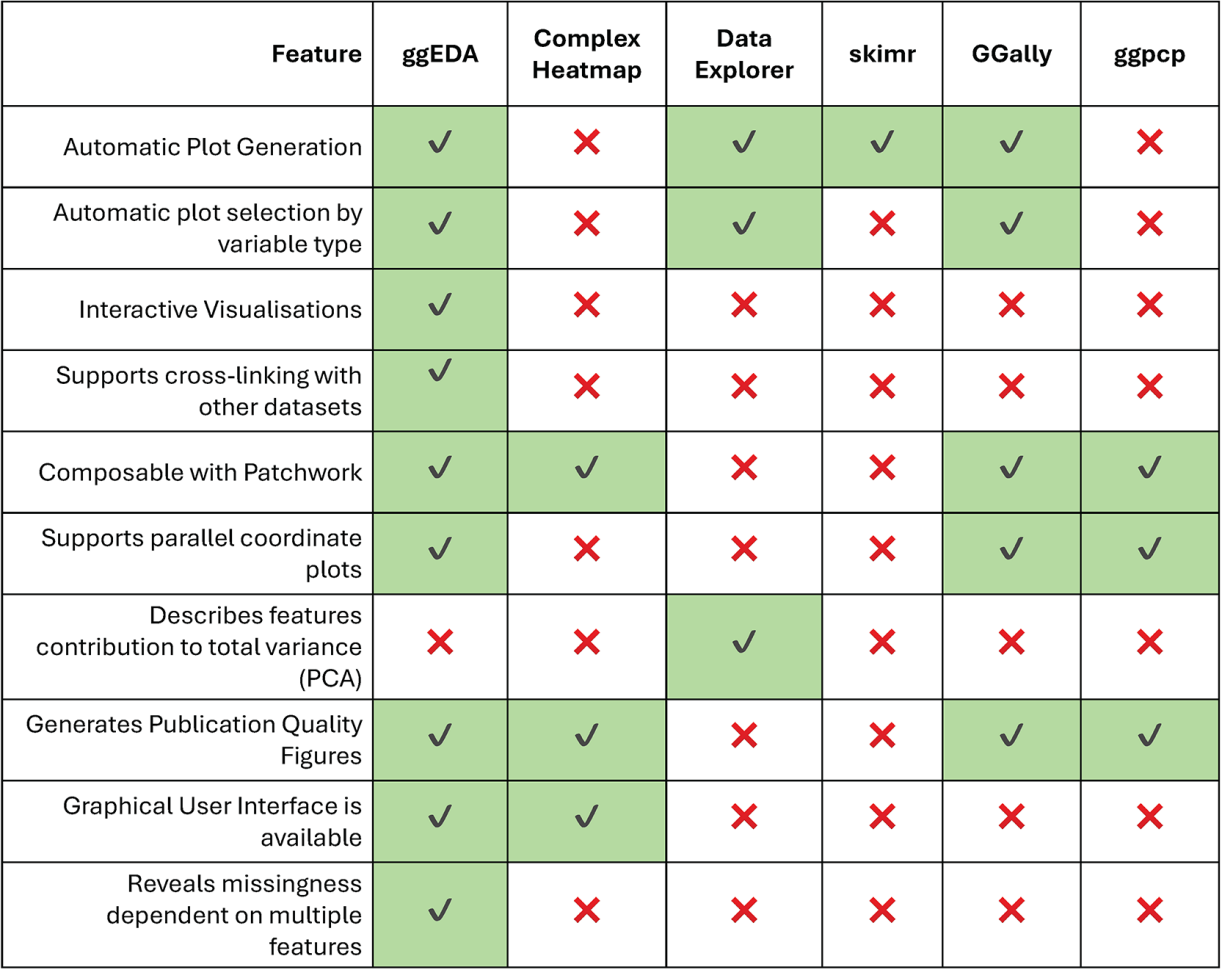
Comparison of R packages that create visualisations commonly used for exploratory data analysis, including ComplexHeatmap, ^1^ Data Explorer, ^2^ skimr, ^3^ GGally ^4^ and ggpcp. ^5^ Due to documented reproducibility issues, ggpcp features could not be verified first-hand.

Exploratory data analysis (EDA) reveals relationships between data features, informing hypothesis generation and downstream analyses. It can also identify data-quality issues such as missingness, bias, and unexpected distribution structure. The R ecosystem already includes popular EDA packages such as **skimr**, which textually summarises completeness and descriptive statistics for individual features (1-dimensional), and **GGally**, which graphically describes pairwise feature correlations (2-dimensional) or multi-feature relationships through PCPs (n-dimensional). **ggEDA** enhances this ecosystem by providing interactive versions of standard n-dimensional visualisations like PCPs and introducing tiled one-dimensional visualisations that more effectively show missingness and relationships between categorical features in smaller datasets. Together, these visualisations provide key advantages over other EDA packages, most notably an ability to reveal a greater variety of multidimensional patterns ( Figure 1).

## Methods

### Implementation

ggEDA is implemented as a standard R package and published on CRAN and the R-universe. The interactiveEDA web app was written using the shiny framework ^6^ and takes ggEDA as a dependency to separate the user-interface codebase from the underlying business logic, which is easier to test. interactiveEDA is compiled into a purely client-side web-assembly app using shinylive ^7^ and hosted as a static web-page on GitHub Pages. Code to produce visualisations is run in the client’s browser instead of a third party server outside the direct control of end-users. The distributed nature of compute also provides scaling benefits compared to traditional server-side shiny apps that quickly slow as concurrent users grow. These security and scalability benefits do come at the cost of slower application startup time.

### Operation

The ggEDA R package can be installed from CRAN ( install.packages( “ggEDA”)). It is compatible with Mac OS X, Windows, and all Unix-like operating systems where R (≥3.5.0) can be installed. Package dependencies are described on the ggEDA CRAN listing .

Data can be explored using the interactiveEDA web app in any modern browser that supports WebAssembly. We performed the most extensive testing in Chrome (version 137.0.7151.120), however the app is also compatible with Firefox, Safari and Microsoft Edge.

## Use cases

To demonstrate how ggEDA and interactiveEDA support exploratory data analysis, we present a series of use cases that highlight their capabilities in visualising multidimensional datasets.

### Creating parallel coordinate plots

**Figure 2.**
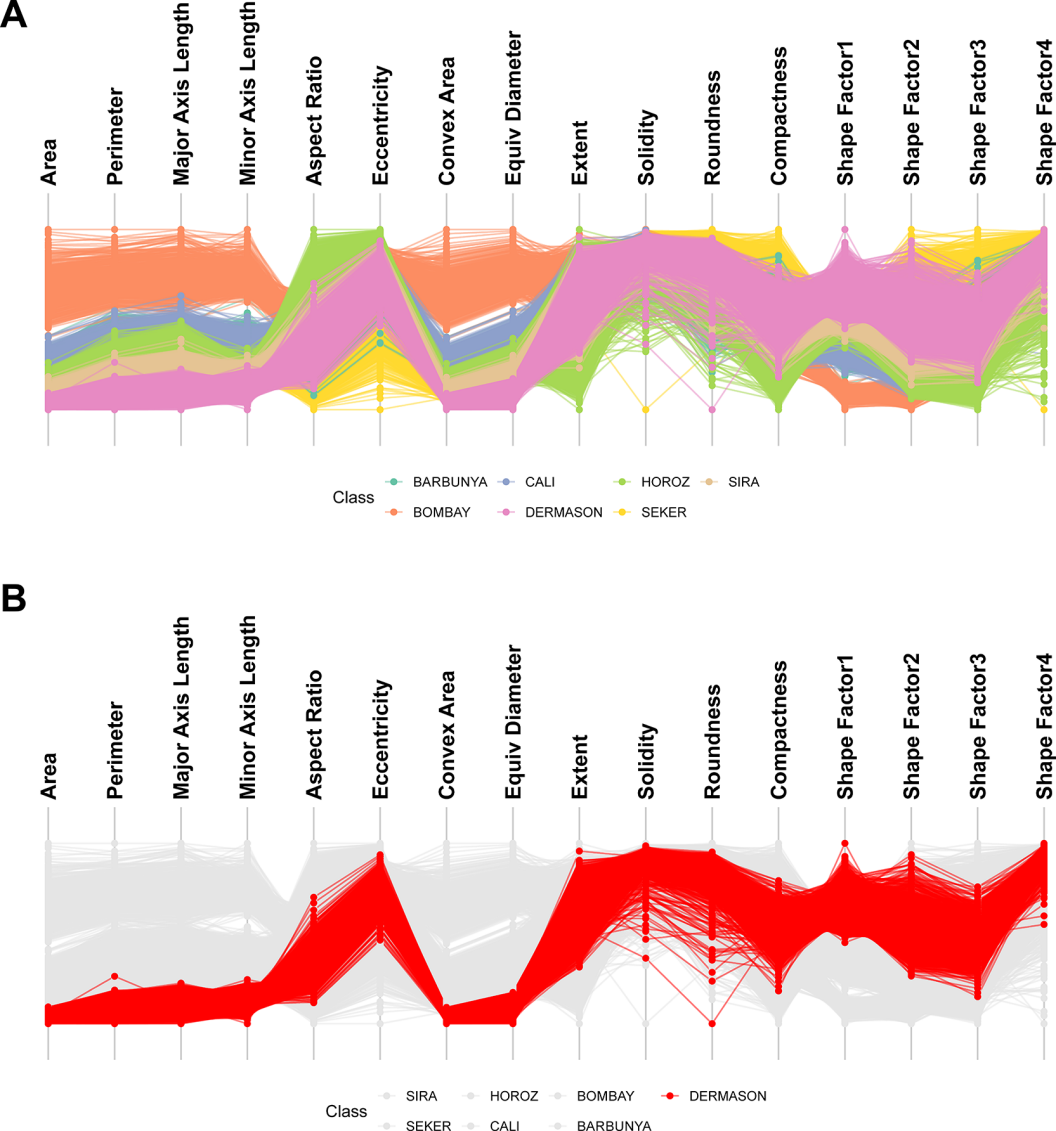
**ggEDA** parallel coordinate plots of the dry beans imaging dataset. ^9^ A) Visualising 16 morphological features of 13,611 grains from common dry bean species reveals clear correlations amongst size-related attributes (Area, Perimeter and Axis Length). Bombay beans were the largest, most convex variety; B) Highlighting a single subclass simplifies both comparison against the full cohort and identification of within-class outliers. For example, Dermason beans (red) are smaller in size than other varieties. One Dermason bean grain had unusually low roundness, highly atypical for this variety.

PCPs are a well-established EDA visualisation that reveal trends in predominantly quantitative datasets and detect outliers in one or more dimensions. Quantitative features are represented as a series of parallel axes with samples visualised as lines passing through each axis at the point of its value. Correlative relationships are revealed when feature axes are ordered based on line crossing minimisation algorithms or mutual information with a categorical feature. ggEDA can produce interactive PCPs from any dataset with quantitative features using the ggparallel(data) command ( Figure 2).

PCPs scale well with large datasets but have several limitations. Visualising the relationships between multiple categorical variables is challenging. Missing data is also difficult to meaningfully represent. For this reason, ggEDA introduces a complementary visualisation composed of vertically aligned tile and bar plots.

### Creating tiled one-dimensional graphics

**Figure 3.**
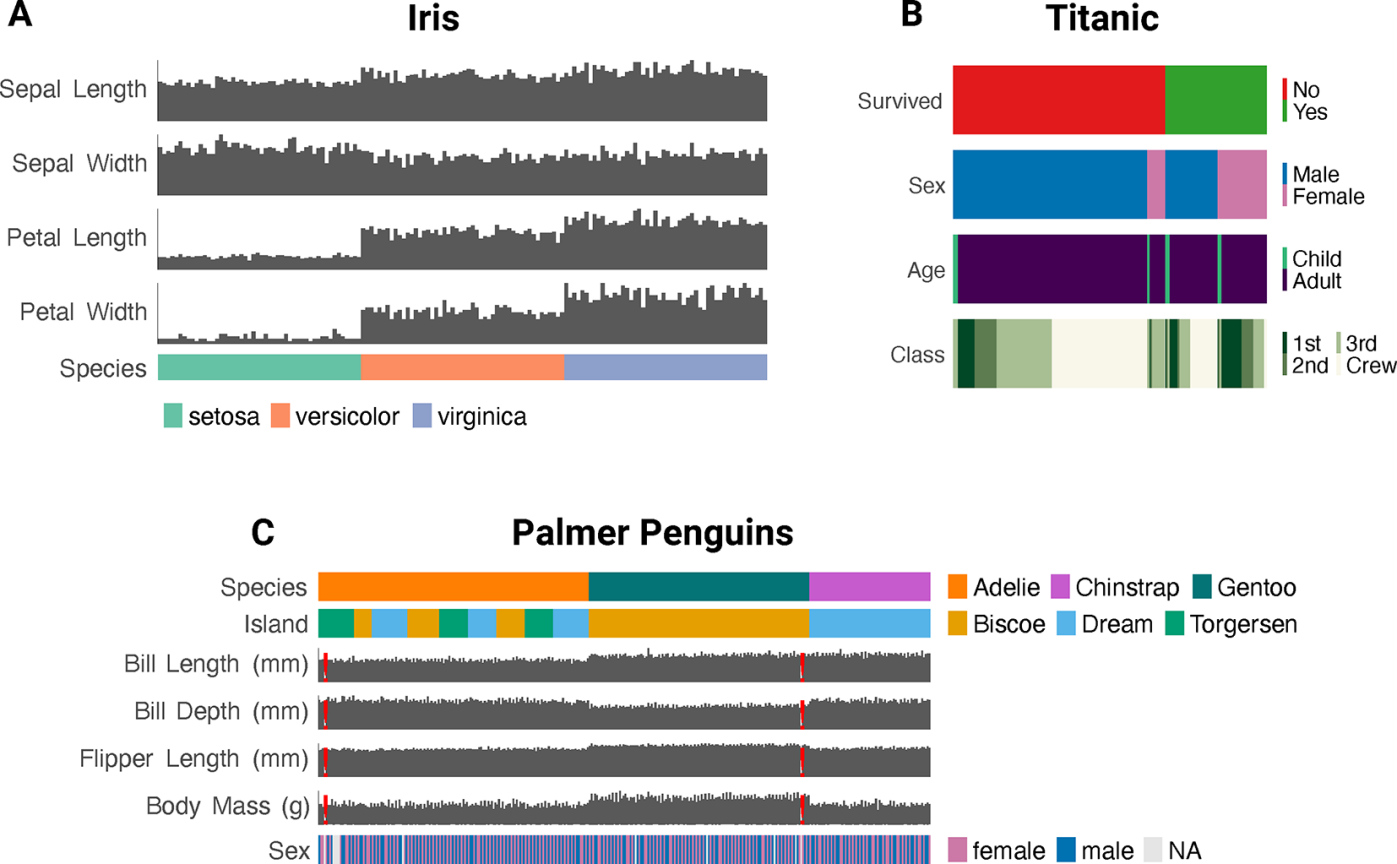
**ggEDA** visualisations of common datasets revealing: A) Petals of the *setosa* species of iris are drastically smaller than other iris species; B) The majority of individuals who perished during the Titanic disaster were adult males; C) *Gentoo* penguins from Biscoe Island have shallower bill depths than *Chinstrap* or *Adelie* penguins, despite their increased body mass.

For small datasets (n < 1000), ggEDA can represent features as distinct, vertically aligned bar or tile plots, with plot types auto-selected based on whether variables are categorical or numeric ( Figure 3).

### Identifying complex multidimensional patterns

**Figure 4.**
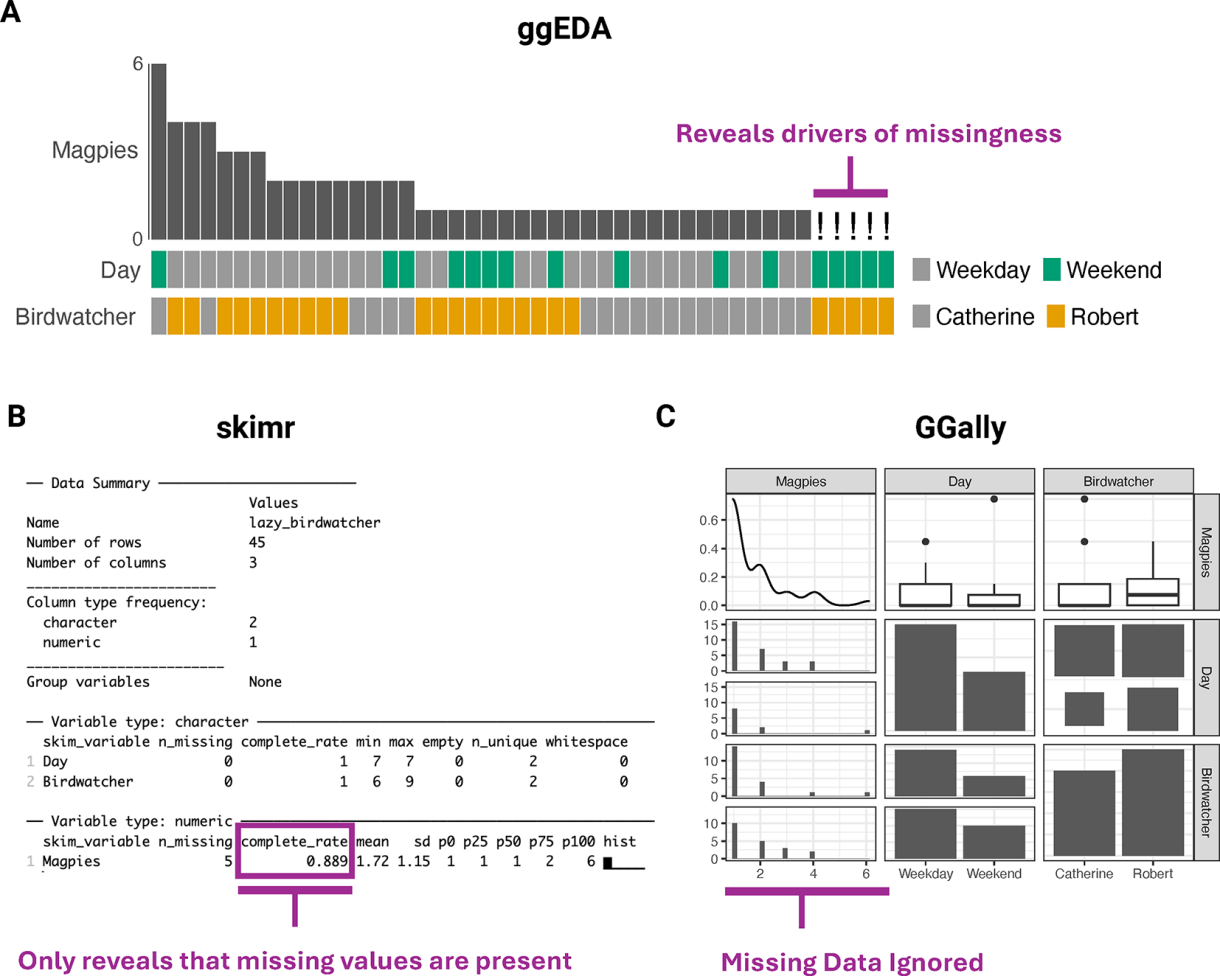
Visualisation of the Lazy Birdwatcher dataset using the **ggEDA** package reveals a pattern of missingness (indicated by exclamation marks) dependent on multiple variables, Birdwatcher and Day (A). This pattern is difficult to detect using one-dimensional EDA tools like **skimr** (B) or two-dimensional tools like ggpairs from the **GGally** package (C).

To demonstrate the advantages of **ggEDA**, we created the artificial *Lazy Birdwatcher* dataset. It describes magpie observations by two birdwatchers, one of whom routinely skips birdwatching on weekends. This introduces a missing data pattern dependent on both the birdwatcher and day of the week. The multidimensional pattern becomes immediately apparent from **ggEDA** stacked tile plots despite being difficult to detect using one-dimensional EDA tools like **skimr**, or two-dimensional tools like **ggpairs** from the **GGally** package ( Figure 4). Despite being n-dimensional, all PCP plot implementations in R also fail to uncover this trend due to either exclusion of missing data or inability to represent clearly the relationships between categorical features.

### Exploring datasets using the interactiveEDA web-app

**Figure 5.**
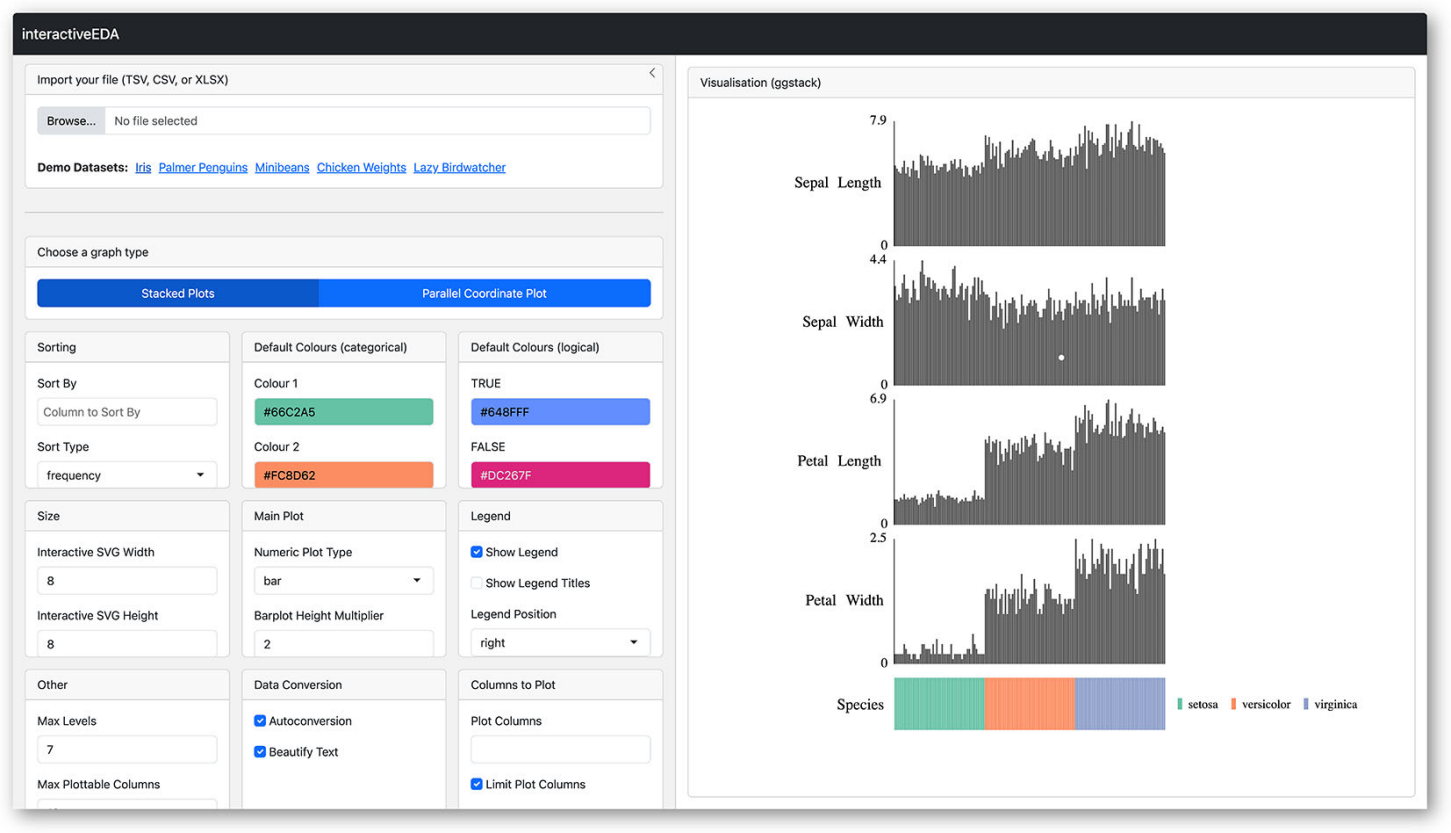
Screenshot of **interactiveEDA**, a web-app providing a graphical user interface for code-free generation of **ggEDA** visualisations.

Despite the advancements provided by ggEDA and other tools in the R ecosystem, a key limitation remains: accessibility for non-programmers, particularly when visualising n-dimensional data. All existing R implementations lack graphical user interfaces ( Figure 1). While shiny web apps offer a potential solution, they often require uploading datasets to external servers, raising privacy concerns. To address these limitations, we developed interactiveEDA, a web-assembly compiled client-side web app for secure, interactive data exploration ( Figure 5). Operating entirely in the browser, interactiveEDA ensures data remains on the user’s machine, increasing ease of use without compromising data privacy. interactiveEDA is available at https://github.com/CCICB/interactiveEDA.

## Summary

ggEDA provides two complementary visualisation strategies for exploratory data analysis: interactive parallel coordinate plots for high-dimensional quantitative data and tiled one-dimensional graphics for exploring missingness and categorical relationships in smaller datasets. These tools help uncover complex patterns and data quality issues with minimal coding. For users without programming experience, the same visualisations are available through the interactiveEDA web app.

## Software availability

**ggEDA:**

• Install using install.packages( “ggEDA”).
• Available from CRAN: https://cran.r-project.org/web/packages/ggEDA/
• Source code: https://github.com/CCICB/ggEDA
• Archived release: https://doi.org/10.5281/zenodo.17290896
• License: MIT

**interactiveEDA:**

• Available at: https://ccicb.github.io/interactiveEDA/
• Source code: https://github.com/CCICB/interactiveEDA
• Archived release: https://doi.org/10.5281/zenodo.17290912
• License: MIT

## Data Availability

Figshare. DryBeans. https://doi.org/10.6084/m9.figshare.29614133.v3 ^8^ This project contains the following underlying data:
• dry_beans.csv: Sourced from the UCI Machine Learning Repository. ^10^ Originally published by Koklu and Özkan in 2020. ^9^ A random subsample (n = 1000) is packaged with ggEDA ( ggEDA::minibeans). Used in Figure 2. • Mini beans.csv Data is available under the terms of the CC BY 4.0 license. Figshare. ggEDA. https://doi.org/10.6084/m9.figshare.30350887.v2 ^11^ This project contains the following underlying data:
• iris.csv: Included with base R. Originally published by Anderson in 1935. ^12^ Used in Figure 3. • titanic.raw.csv: Loaded from the datarium R package. ^13^ Originally published by the British Board of Trade in 1990. ^14^ Used in Figure 3. • penguins.csv: Loaded from the palmerpenguins R package. ^15^ Originally published by Gorman *et al.* in 2014. ^16^ Used in Figure 3. • lazy_birdwatcher.csv: Artificial dataset bundled with the ggEDA R package ( ggEDA::lazy_birdwatcher). Used in Figure 4. Data is available under the terms of the CC0 license.
