# Supplementary material for: ggEDA: Visualisations for exploratory data analysis using tiled one-dimensional graphics and parallel coordinate plots
Source: F1000Res. 2025 Nov 13;14:1248. [Version 1] doi: 10.12688/f1000research.168305.1 (PMC13096783; doi:10.12688/f1000research.168305.1)
Supplement: Peer review history

# Reviewer report: Reviewer response for version 1

**DOI:** 10.5256/f1000research.185479.r473463

**Published:** 2026-04-20

**Authors:** Maria Cristina Ferreira de Oliveira

**Affiliations:** 1 Computer Science, Universidade de Sao Paulo, São Carlos, State of São Paulo, Brazil

The paper introduces a novel exploratory data analysis package for the R ecosystem. The package, named ggEDA, that incorporates two interactive visualizations for multidimensional data: tiled 1-dimensional bar plots and parallel coordinate plots. 

 Implementation details and installation instructions are provided in the text.  Use cases for typical datasets are provided to illustrate the techniques' use.  A web-based application is also provided for non-programmers. 

 The contribution nicely complements the facilities already available in R, and the authors clearly express the advantages and limitations, e.g., regarding the scalability of the techniques.

Are the conclusions about the tool and its performance adequately supported by the findings presented in the article?

Yes

Is the rationale for developing the new software tool clearly explained?

Yes

Is the description of the software tool technically sound?

Yes

Are sufficient details of the code, methods and analysis (if applicable) provided to allow replication of the software development and its use by others?

Yes

Is sufficient information provided to allow interpretation of the expected output datasets and any results generated using the tool?

Yes

Reviewer Expertise:

computer science, data visualization, data mining.

I confirm that I have read this submission and believe that I have an appropriate level of expertise to confirm that it is of an acceptable scientific standard.

# Reviewer report: Reviewer response for version 1

**DOI:** 10.5256/f1000research.185479.r472021

**Published:** 2026-04-10

**Authors:** Valeria Velásquez-Zapata

**Affiliations:** 1 GreenLight Biosciences, Durham, NC, USA

The article introduces ggEDA, an R package designed to streamline exploratory data analysis (EDA) by providing two complementary visualization strategies: interactive parallel coordinate plots (PCPs) and tiled one-dimensional graphics. By automating complex visualizations, ggEDA helps users analyze multidimensional patterns and data quality issues that might otherwise require extensive manual review or statistical modeling.

 To increase accessibility for non-programmers, the authors developed interactiveEDA, a web-based application built using the a shiny framework. Overall, ggEDA and interactiveEDA together provide a powerful and user-friendly suite for multidimensional data exploration. The authors also provide example datasets so users can explore the functions.

Are the conclusions about the tool and its performance adequately supported by the findings presented in the article?

Yes

Is the rationale for developing the new software tool clearly explained?

Yes

Is the description of the software tool technically sound?

Yes

Are sufficient details of the code, methods and analysis (if applicable) provided to allow replication of the software development and its use by others?

Yes

Is sufficient information provided to allow interpretation of the expected output datasets and any results generated using the tool?

Yes

Reviewer Expertise:

Bioinformatics

I confirm that I have read this submission and believe that I have an appropriate level of expertise to confirm that it is of an acceptable scientific standard.
